# Supplementary material for: SOX9 and SEMA7A regulate cell plasticity in the postpartum mammary gland with implications for breast cancer
Source: bioRxiv. 2026 May 18:2026.05.14.725155. Preprint. [Version 1] doi: 10.64898/2026.05.14.725155 (PMC13228434; doi:10.64898/2026.05.14.725155)
Supplement: Supplement 2 [file media-2.pdf]

Supplemental Table 1: Selected cell segmentation features for Neural Network training. Cellular segmentation features used to train the neural network to separate stromal and epithelial cells. DAB staining measurements were excluded to prevent classifying decisions based on the presence or absence of our SOX9 stain.

**Feature**	**Selected (True/False)**
Area µm^2	True
Length µm	True
Circularity	True
Solidarity	True
Max diameter µm	True
Min diameter µm	True
Hematoxylin: Mean	True
Hematoxylin: Median	True
Hematoxylin: Min	True
Hematoxylin: Max	True
Hematoxylin: Std.Dev	True
DAB: Mean	False
DAB: Median	False
DAB: Min	False
DAB: Max	False
Smoothed 25 µm: Area µm^2	True
Smoothed 25 µm: Length µm	True
Smoothed 25 µm: Circularity	True
Smoothed 25 µm: Solidarity	True
Smoothed 25 µm: Max diameter µm	True
Smoothed 25 µm: Min diameter µm	True
Smoothed 25 µm: Hematoxylin: Mean	True

Smoothed 25 µm: Hematoxylin: Median	True
Smoothed 25 µm: Hematoxylin: Min	True
Smoothed 25 µm: Hematoxylin: Max	True
Smoothed 25 µm: Hematoxylin: Std.Dev	True
Smoothed 25 µm: DAB: Mean	False
Smoothed 25 µm: DAB: Median	False
Smoothed 25 µm: DAB: Min	False
Smoothed 25 µm: DAB: Max	False
Smoothed 25 µm: Nearby detection counts	True
Smoothed 50 µm: Area µm^2	True
Smoothed 50 µm: Length µm	True
Smoothed 50 µm: Circularity	True
Smoothed 50 µm: Solidarity	True
Smoothed 50 µm: Max diameter µm	True
Smoothed 50 µm: Min diameter µm	True
Smoothed 50 µm: Hematoxylin: Mean	True
Smoothed 50 µm: Hematoxylin: Median	True
Smoothed 50 µm: Hematoxylin: Min	True
Smoothed 50 µm: Hematoxylin: Max	True
Smoothed 50 µm: Hematoxylin: Std.Dev	True
Smoothed 50 µm: DAB: Mean	False
Smoothed 50 µm: DAB: Median	False
Smoothed 50 µm: DAB: Min	False
Smoothed 50 µm: DAB: Max	False
Smoothed 50 µm: Nearby detection counts	True
